# Associations between Park and Playground Availability and Proximity and Children’s Physical Activity and Body Mass Index: The BEACH Study

**DOI:** 10.3390/ijerph19010250

**Published:** 2021-12-27

**Authors:** Javier Molina-García, Cristina Menescardi, Isaac Estevan, Ana Queralt

**Affiliations:** 1 Department of Teaching of Musical, Visual and Corporal Expression, University of Valencia, Avda. dels Tarongers, 4, 46022 Valencia, Spain; cristina.menescardi@uv.es (C.M.); isaac.estevan@uv.es (I.E.); 2 AFIPS Research Group, University of Valencia, 46010 Valencia, Spain; ana.queralt@uv.es; 3 Department of Nursing, University of Valencia, Jaume Roig, s/n, 46010 Valencia, Spain

**Keywords:** parks, green spaces, playgrounds, physical activity, active commuting to school, obesity, body mass index, walkability, neighborhood, environment design

## Abstract

A cross-sectional study was designed to evaluate the relationship between the availability and proximity to parks and playgrounds and physical activity (PA). Moreover, the accessibility to parks and playgrounds and its association with active commuting to/from school (ACS) and body mass index (BMI) were analyzed. The sample was composed of children aged 6–12 years old from the BEACH (Built Environment and Active CHildren) study in Valencia, Spain. The availability and proximity to parks and playgrounds were calculated at different buffer sizes (250, 500, 1000 and 1250 m) using geographical information system data. PA out of school was assessed using accelerometers. Sociodemographics and ACS were measured with a parent questionnaire. Objectively measured weight and height were used to calculate BMI. Mixed linear regression analyses were conducted for each exposure variable, adjusting for sociodemographics, neighborhood walkability level, and participant clustering. The number of parks and playgrounds were positively associated with moderate to vigorous PA (MVPA) and total PA (TPA); including light PA and MVPA, during weekdays, in different buffer sizes. A negative relationship between distance to the nearest playground and TPA during weekdays was found. In addition, the number of playgrounds was positively related to ACS in different buffer sizes, whereas park land area was negatively related to the BMI percentile. This study highlights the importance of assessing the availability and proximity to parks and playgrounds in children’s neighborhoods when PA behavior and weight status are analyzed. Study findings may help policymakers when targeting interventions to promote health-enhancing behaviors in children.

## 1. Introduction

The role of the built environment on children’s health has attracted great attention in the literature during the last decade [1,2]. Nevertheless, there is little consensus about the impact of the built environment on health-related outcomes for children [1,3]. This is mainly due to the inconsistency in approaches and measures used to evaluate built environment for youth [1,3]. According to current evidence, a child-friendly built environment has high walkability levels (e.g., well-connected streets) and provides access to recreational facilities (e.g., parks and playgrounds), that have been positively associated with physical activity (PA), including active commuting to/from school (ACS) and recreational play [1,4,5,6,7,8,9,10,11]. Furthermore, a positive built environment has a significant role in preventing non-communicable diseases reducing obesity rates, diabetes or coronary heart disease [1,4,12]. Although the causes of obesity are multifactorial, built environmental factors can potentially drive obesity-related behaviors [12]. The neighborhood built environment has significantly links to children’s behaviors, such as PA and sedentary lifestyles [13]. Thus, built environment is considered a key determinant of health, including obesity, and lifestyle in childhood [12,14,15].

The majority of studies about the impact of built-environment attributes on children’s health come from the USA, UK, Canada, Australia and New Zealand [1]. Southern European countries, such as Spain, show the highest rates of obesity among children in Europe [16] and one of the highest levels of physical inactivity [17], hence, the importance of analyzing the built environment in relation to children’s health with the aim to design more efficient health policies.

Public neighborhood parks are considered key locations where children can play outdoors and safely in urban areas [18,19,20]. Parks include specific attributes and features that can be important to encourage PA in different age groups. The literature indicates that the presence of playgrounds is one of the most attractive park features to the population of children [20,21,22,23,24]. A playground is usually an outdoor area designed for children to play and is located in public parks or other public open spaces. Globally, public parks and playgrounds can have a prominent role in reducing physical inactivity and obesity among children [1].

The influence of an activity-friendly neighborhood on PA behavior can be different depending on the type of day of the week (i.e., weekdays vs. weekend days) [25]. Moreover, children accumulate more PA on weekdays compared to weekend days [26]. That is why it is important to analyze PA by differentiating the type of day when analyzing the association between the built environment and PA in young people. However, there is a lack of studies examining this association considering the weekdays and the weekends separately.

Previous evidence linked park and playground spaces with health-related outcomes for children. Nevertheless, to our knowledge, there are no previous studies carried out in Spain analyzing the relationship between the accessibility to parks/playgrounds and PA and body mass index (BMI) among children. Thus, the main purpose of this study was to examine the availability and proximity to parks and playgrounds in the home neighborhoods and its association with PA behaviors among children in Valencia, Spain. Out-of-school PA, during weekdays and weekend days, was analyzed. Moreover, the accessibility to parks and playgrounds and its association with ACS and BMI were analyzed. Finally, differences in outcomes between children living in neighborhoods with high and low values for significant park and playground attributes were examined.

## 2. Materials and Methods

### 2.1. Study Design, Procedure and Participants

This study is based on an observational cross-sectional study design. Data for this study were collected as part of the Built Environment and Active Children (BEACH) study in the city of Valencia, Spain. The data collection was carried out in February–May 2018. This data collection constituted a pilot study in order to evaluate the feasibility, duration and possible adverse events before executing the full-scale BEACH study. The BEACH study uses the methodology of the International Physical Activity and the Environment Network (IPEN) study carried out in adolescents in the city of Valencia [25,27]. The IPEN study has developed an international set of protocols aimed at maximizing the comparability of built environment features and PA outcomes between different geographical contexts [27,28]. The main aim of the BEACH study is to analyze the associations of personal, psychosocial and built environment variables with PA and sedentary behavior (accelerometer-measured and self-reported) and anthropometric measures among school-age children in Valencia, Spain.

A primary education school was selected by convenience sampling, as the investigator (second author) was teaching at the school at the time of the study. Following IPEN methodology [25,27], the socio-economic status (SES) of the school neighborhood (i.e., census block) was determined. The selected school was located in a high-SES neighborhood using educational level as SES indicator. Primary education in Spain consists of six academic school years, and students are between the ages of 6 and 12 years. All the students from the six school years were invited. To this end, different informational meetings were organized at the school to present the study to parents or legal guardians. An initial sample of 95 students agreed to participate in the study (response rate of 36%). Based on inclusion criteria, 3 participants were excluded from the study. Inclusion criteria were: children aged 6–12 years; living in the city of Valencia; and being able to walk without assistance. Moreover, 9 children were excluded because their questionnaires had missing data or incomplete/inaccurate postal addresses. Thus, 83 children (aged 6–12 y; 43 girls) were included in the final sample.

### 2.2. Measures

#### 2.2.1. Exposure: Availability and Proximity to Parks and Playgrounds in the Neighborhood

**Figure 1:**
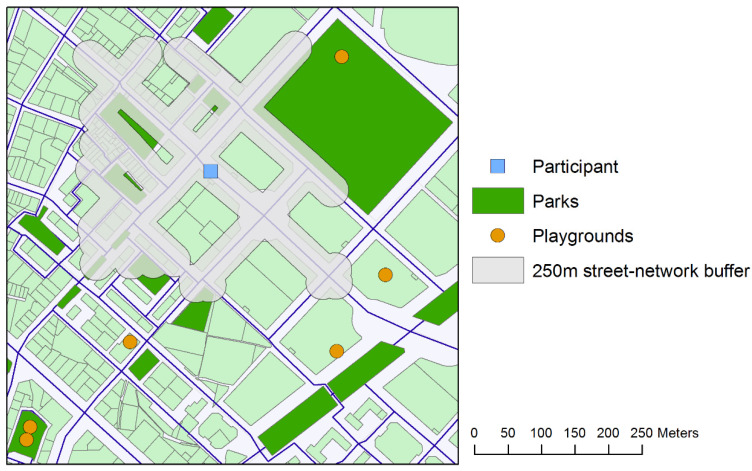
Example of the accessibility to parks and playgrounds in a participant’s neighborhood using a 250 m street-network buffer.

The availability and proximity to parks and playgrounds in the home neighborhood were generated with ArcGIS 10.2 software (ESRI Inc., Redlands, CA, USA). Different street-network buffers (i.e., 250, 500, 1000 and 1250 m) were defined to estimate accessible environmental features according to previous research in youth samples [27,29,30]. The street-network distance of 1250 m is considered as that from which the number of passive commuters exceeds the number of active commuter to school in Spanish urban children [30]. Active commuting involves active modes of transport such as walking or cycling, whereas passive commuting involves passive ones (e.g., car, motorbike or public transport). In the present study, we used this threshold distance to define the largest street-network buffer. The following variables were used in our analyses: park land area contained within or intersected by buffer, number of parks contained or intersected by buffer, street-network distance to the nearest park, number of playgrounds contained or intersected by buffer, and street-network distance to the nearest playground. Figure 1 shows an example of the location of parks and playgrounds in a participant’s neighborhood using a 250 m street-network buffer. The spatial distribution of park land use and the location of playgrounds were obtained from the OSM (Open Street Maps) service. Additionally, we used the General Urban Plan of the city of Valencia (https://www.valencia.es/dadesobertes/es/data/, accessed on 20 March 2020) to verify the data obtained from the OSM.

#### 2.2.2. Study Outcomes: PA, ACS and BMI

PA was objectively measured with ActiGraph GT3X+ accelerometers (Actigraph, Pensacola, FL, USA). These instruments have been shown to be valid and reliable in youth [31]. Parents were instructed to have children wear the accelerometer for 7 days, except during sleep and during water-based activities. ActiLife 6 (ActiGraph, Pensacola, FL, USA) software was used to process the accelerometer data, which were aggregated to 15 s epochs. A valid day was one in which the accelerometer was worn for a minimum of 10 h per weekday or 8 h per weekend day. Non-wear time was considered strings of 60 or more minutes of 0 counts. Children had to have at least 4 valid days, including at least 1 weekend day. Only one child in the final sample did not have valid accelerometry data. PA was scored using the Evenson cutoff points [32] to calculate the amount of time spent in light PA (101 to 2295 counts per 60 s epoch); moderate PA (2296 to 4011 counts per 60 s epoch); and vigorous PA (≥4012 counts per 60 s epoch). Then, these types of physical activities were grouped into two categories: moderate to vigorous PA (MVPA) and total PA (TPA), including light PA and MVPA. In the present study, we used out-of-school PA because this activity is the one that can be influenced by the built environment in which the children live. Moreover, considering previous research, we analyzed PA while differentiating between weekdays and weekend days [25,26]. Thus, three types of variables were created for both MVPA and TPA: “per day”, average PA considering all days of the week; “per weekday”, average PA considering only the days from Monday to Friday; and “per weekend day”, average PA taking into account Saturday and Sunday.

A paper questionnaire completed by parents was used to assess the modes of transport to and from school. This instrument includes the following question: “In an average school week, on how many days do you use the following modes of transportation to get to and from school?” This question was adapted from the Centers for Disease Control [33] by IPEN study investigators [27]. The response options were walk, bike, skateboard, public transit, school bus and car. The total number of trips per week traveled by walking, cycling or skateboarding was obtained (range = 0–10 trips). This question had previously been satisfactorily used among youth samples in previous research and had good reliability [34,35].

Height and weight were directly measured following standardized protocols by using a stadiometer scale (SECA, Hamburg, Germany) and a bioelectrical impedance scale (Tanita BC-601, Japan). Then, BMI (kg/m^2^) was calculated. The CDC growth charts [36] were used to calculate BMI percentile (age and sex-adjusted) and weight-status category (i.e., “overweight”, BMI ≥ 85th to 95th percentile; and “obesity”, BMI ≥ 95th percentile). Only two participants were categorized as underweight (i.e., BMI < 5th).

#### 2.2.3. Covariates

Demographic covariates assessed by the parent questionnaire were participant gender, age and SES (highest parental education). The parents’ level of education ranged from 1 (none) to 6 (university training). The street-network distance from participants’ residence to school was calculated using Geographic Information System (GIS) procedures. Participants’ home neighborhoods were classified according to walkability. Census blocks, as the smallest administrative unit, was used to delineate home neighborhoods. The city of Valencia was divided into almost 600 census blocks [25]. Following the IPEN protocol [27], GIS-based neighborhood walkability was calculated for each census block group by summing the z-scores of three built environment measures: intersection density, net residential density and land use mix [37]. Census blocks were divided into deciles based on their walkability index values. Then, census blocks were categorized as “high walkability” (highest five deciles) or “low walkability” (lowest five deciles).

### 2.3. Data Analysis

Descriptive statistics (e.g., means, standard deviations, skewness for continuous measures, frequencies and percentages) were calculated to analyze the distributions of the measurements. The normality of the data was checked using Kolmogorov–Smirnov test. Mixed linear regression analyses (SPSS MIXED procedure) assessed the relationship of each exposure variable (availability and proximity to parks and playgrounds in the neighborhood at different buffer sizes) with outcomes (PA, ACS and BMI), adjusting for all covariates, including neighborhood walkability level, as fixed effects and participant clustering in census block groups as a random effect. Mixed linear regression analyses are an extension of simple linear regression analyses to allow both fixed and random effects [38]. The statistical assumptions of a mixed-effects model involve, among other things, independence of the random effects vs. covariates. We show *β* estimates and *t* statistics (and significance levels) from the adjusted mixed models. The confidence intervals and effect sizes are not reported because of space limitations in the tables. Quartiles for exposure variables were also calculated to compare extremes. Differences in outcomes between participants with high (Q1) and low values (Q4) for significant park and playground attributes were presented in a table. The Mann–Whitney U test was used to compare continuous measurements, and the chi-square test was used to compare proportions (i.e., % Overweight/obesity) between the two quartile groups. Mann–Whitney U tests were used because of the small sample size in each of the quartile groups. Data were analyzed using SPSS v. 26.0 (SPSS, Chicago, IL, USA). Statistical significance was set at *p* = 0.05.

## 3. Results

Table 1 shows socio-demographic characteristics and physical activity behaviors of the sample. The children resided in 29 different census blocks in the city of Valencia. Our study found that 77.1% of the children resided in high-walkability neighborhoods. The 71.6% of the families had a mother/parent with a university degree or university diploma program.

Table 2 presents the study descriptive statistics of the availability and proximity to parks and playgrounds variables for all participants using different buffer sizes. In the present study, 54.5% of the playgrounds were located in parks, whereas the rest of playgrounds were located in other public open spaces such as squares, wide sidewalks or other green spaces (e.g., gardens).

Table 3, Table 4 and Table 5 present the mixed-model regression results. The number of parks was positively associated with MVPA during weekdays in a 1000 m buffer, and the number of playgrounds was positively related to MVPA during weekdays in the 500, 1000 and 1250 buffers (Table 3). The comparison of the two extreme quartiles (i.e., Q1 and Q4) for park and playground attributes in relation to the study outcomes is shown in Table 6. In this regard, children living in neighborhoods in the Q1 (highest values) for the number of playgrounds could achieve 7 min more MVPA (per day) than those living in neighborhoods in the Q4 (lowest values). As shown in Table 4, the number of parks (for 500 and 1000 m buffers) and number of playgrounds (for all buffer sizes) were positively associated with TPA during weekdays, whereas a negative association between distance to the nearest playground and TPA (during weekdays) was found. Children living in neighborhoods in the Q1 for the number of playgrounds could achieve 22 and 35 min more TPA per weekday and per day, respectively, than those living in neighborhoods in the Q4 (Table 6). Additionally, children who lived farthest from the nearest playground (Q1) achieved 14 min less TPA than those who lived in neighborhoods in the Q4 (Table 6). We also found a positive relationship between the number of playgrounds and ACS in the 250 and 500 m buffers (Table 5). Children living in neighborhoods in the Q1 for the number of playgrounds could actively travel 5 trips/week more than those living in neighborhoods in the Q4 (Table 6). In addition, park land area was negatively related to BMI percentile in the 250 and 500 m buffers (Table 5).

## 4. Discussion

In this study, the availability and proximity to parks and playgrounds in the home neighborhood were found to be significantly associated with objectively measured PA and BMI percentile in Spanish children. One of the main findings of the present study is that the number of parks and playgrounds has been positively related to PA (i.e., MVPA and TPA) on weekdays but not on weekends. These findings are consistent with previous research where the influence of the built environment attributes on PA behavior can be different depending on the type of the day [25]. A possible explanation of the lack of association on weekend days is that PA behavior can be influenced by other environmental attributes, both macro- and micro-scale factors, such as quality/safety of parks and playgrounds and the presence of sports facilities in the neighborhood [1,39,40,41]. Furthermore, children may spend additional time out of the home neighborhood on weekends. In addition, children accumulate less PA on weekend days in contrast to weekdays because weekend days tend to be less structured [26]. Future intervention studies should consider analyzing other built environmental variables that can affect PA on weekend days. Additional studies in future should be performed focusing on parental perceptions of the neighborhood environment that can affect children’s outdoor playtime depending on the type of the day [42].

This study also found that the park availability was significantly associated with higher levels of MVPA and TPA during weekdays in the 500 m and 1000 m buffers. Our findings are in line with previous evidence from other countries [1,3,5,11,22] and strengthen the importance of designing neighborhoods where park availability should be high to promote children’s health. According to the review performed by Ding et al. [3], there was a positive association between the density of nearby parks or proximity to parks and PA in 42% of results in children. Interestingly, in this study, although significant associations were found between both the number of parks and playgrounds, and PA, the associations are more significant with the number of playgrounds. Moreover, the number of playgrounds was positively related to PA during weekdays in the four buffer sizes. It seems that these type of play facilities would favor the PA to a greater extent. As previously indicated, children living in the neighborhoods (1250 m street-network buffer) with the highest number of playgrounds could achieve 22 min more of TPA per weekday. As is known, the availability of playgrounds can be positively related to PA in children [20,21,22,23]. Our findings concur with a previous Australian study [21], showing that the upgrade of a park, including an all-abilities playground (among other park improvements), was related to an increase in park visitation among children. Nevertheless, another study in Australia [43] found no associations between the refurbishing of parks (including the addition of new playgrounds) and park visitation or MVPA in children. Thus, additional similar natural experiment studies, in multiple countries and cities, will be required to better understand the effect of the inclusion of playgrounds on park-based PA. Future research should also examine the park features and their characteristics that are attractive to children [40,41].

Considering our data, one interesting observation is that the playgrounds were not predominantly located in parks. Future studies that focus on the influence of built environment on children’s health should evaluate the different open spaces (e.g., squares, wide sidewalks, or other green spaces) in which PA takes place, as well as the availability or proximity to natural environments, including both green and blue spaces [1].

Distance from home to the nearest park was not related to PA in the present study. A study carried out by Dunton et al. [44] in US children and adolescents showed a significant increase in park use as distance from home decreased. Nevertheless, previous research from Australia [45,46] has shown that children do not always visit the nearest park. These previous studies indicated that some children travelled farther distances with their parents to other parks, even though there was a closer park. It seems that the park attractiveness would be more related to the park use than the distance. Furthermore, current evidence shows that the presence of certain features within parks, such as playgrounds, is related with PA among children [20,21,22,23,24]. In line with this idea, a study from Australia [47] indicated that the presence of playgrounds at the nearest public open space to home was positively related to children’s MVPA. In our case, the distance from home to the nearest playground influenced weekday TPA, which included not only MVPA but also light PA. Children who lived closest from the nearest playground (mean distance = 62.1 m) achieved about 14 min more TPA than those who lived farthest (mean distance = 306.6 m). Another study from US [48] showed that having a playground within 800 m from child’s home increased the probability by 2.5 times of meeting MVPA recommendations. In the current study, it is notable to highlight that distance to the closest playground was significantly related to TPA but not to MVPA. Hence, it is important to analyze in the future what are the playground characteristics that promote high intensity levels of PA in children.

Additionally, our data demonstrated that the number of playgrounds in the home neighborhood was positively associated with ACS in the smaller buffers (250 m and 500 m). Our results highlight that children who resided in neighborhoods with the highest number of playgrounds could actively travel five trips/week more than those residing in neighborhoods with fewer playgrounds. It is notable that the mean distance to school for children within the study was only 680 m. This distance is well below the threshold distance that best discriminated walkers from passive commuters in Spanish urban children [30] (i.e., 1250 m). Thus, most of the participants lived within a walkable distance to school. Children may use the playgrounds on the routes to and from school. These results support the idea that an activity-friendly neighborhood should be designed to be attractive to youth, including the availability of activity-promoting destinations and public open spaces that allow outdoor play [1,49]. In addition, it will be relevant to develop future investigations that evaluate the real routes to school (e.g., using Global Positioning Systems), as well as the macro- and micro-scale environmental factors of the neighborhood that can make it more attractive to children.

In the present study, there was a negative relationship between park land area and BMI percentile in the 250 and 500 m buffers, but not in the larger buffer sizes. According to our findings, the 500 m distance would be the neighborhood size to be used to find significant associations between the availability of parks with youth obesity. The 500 m distance would correspond to an approximately 5- to 10-minute walk [11]. Our findings are consistent with previous research that reported that availability of parks and sport/recreation public open spaces was related to reductions in youth obesity [1,50,51]. A study in US found a considerable variation in associations between park availability and child obesity by gender, SES and race/ethnicity [52]. Future studies should examine if these socio-demographics moderate the association between park availability and children obesity in other geographic contexts. Likewise, it is relevant to bear in mind that an obesogenic environment promotes not only physical inactivity but also unhealthy diets [53,54]. For this reason, the relationship between the availability of parks and children obesity should be analyzed by taking into account the food-related environment in the future. For instance, the availability of fast food stores or the lack of fruits and vegetables stores are aspects of an obesogenic environment [55]. Thus, a multidisciplinary approach is necessary to address the complexity of built environment influences on children obesity, including experts from diverse scientific fields.

### Strengths and Limitations

One strength of the study was the use of objective measures to examine built environment attributes (i.e., parks and playgrounds variables and walkability levels), PA out of school and BMI. Moreover, the study setting in Valencia added geographic and cultural diversity to prior studies. Another strength was the evaluation of different street-network buffer sizes. However, there are different limitations. Our data were from a pilot study with a small sample size recruited from a single school; thus, we must be cautious when interpreting the findings in terms of generalizability. The cross-sectional design was another important limitation. Longitudinal follow-up studies and natural experiments are necessary in the future. Finally, this study did not include the evaluation of the accessibility to other recreational and sports facilities such as sports centers and free outdoor sports courts in the children’s neighborhood. Additionally, a quality/safety measurement of parks and playgrounds was not performed.

## 5. Conclusions

The number of parks and playgrounds were positively associated with PA during weekdays in different buffer sizes. The number of playgrounds was positively related to ACS in different buffer sizes, whereas park land area was negatively related to the BMI percentile. Our study adds evidence to the relationship between built environment and children’s health. In particular, this study highlights the importance of assessing the availability and proximity to parks and playgrounds in children’s neighborhoods when PA behavior and weight status are analyzed. Study findings may help policy-makers when targeting interventions to promote health-enhancing behaviors in school-age children. A health-supportive neighborhood for children should provide reasons to be actively playing outside, including the availability of parks and playgrounds. It is necessary to convince policy-makers and politicians to preserve green and open spaces in cities.

## Figures and Tables

**Table 1:** Descriptive statistics of the sample.

	Range	Mean (SD) or %
Gender (female)	-	51.8
Age	6–12	8.78 (1.69)
Socio-economic status (highest parental education)	1–6	5.32 (1.18)
Distance to school from home (m)	32.12–4537.53	680.36 (907.05)
BMI percentile (age and sex-adjusted)	0.30–99.50	61.48 (28.62)
Overweight and obesity ^1^	-	28.9
MVPA (min per day)	12.16–107.81	44.06 (19.40)
TPA (min per day)	97.47–355.23	243.39 (51.70)
ACS (trips per week; 98% walk trips)	0–10	8.37 (3.53)

MVPA: moderate to vigorous physical activity. TPA: total physical activity. ACS: active commuting to/from school. ^1^ BMI at or above the 85th percentile.

**Table 2:** Descriptive statistics of the park and playground variables for all study participants.

	250 m Street Buffer	500 m Street Buffer	1000 m Street Buffer	1250 m Street Buffer
	Mean (SD)	Mean (SD)	Mean (SD)	Mean (SD)
Park land area (m^2^)	42,841.75 (170,241.57)	111,545.23 (175,772.78)	343,741.53 (262,046.88)	826,958.87 (527,667.76)
Number of parks	3.41 (2.80)	9.05 (4.92)	27.63 (7.76)	36.81 (8.35)
Street-network distance to nearest park ^1^ (m)	196.31 (139.16)			
Number of playgrounds	4.45 (2.86)	11.17 (3.94)	27.52 (6.15)	35.10 (5.57)
Street-network distance to nearest playground ^1^ (m)	164.54 (97.89)			

^1^ Not dependent on buffer size.

**Table 3:** Mixed-model regression results for the relationship between the availability and proximity to parks and playgrounds in the participants’ neighborhood and moderate–vigorous physical activity (MVPA).

	250 m Street Buffer						500 m Street Buffer						1000 m Street Buffer						1250 m Street Buffer					
	MVPA (Per Day)		MVPA ^1^ (Weekday)		MVPA (Weekend Day)		MVPA (Per Day)		MVPA ^1^ (Weekday)		MVPA (Weekend Day)		MVPA (Per Day)		MVPA ^1^ (Weekday)		MVPA (Weekend Day)		MVPA (Per Day)		MVPA ^1^ (Weekday)		MVPA (Weekend Day)	
	*β*	*t*	*β*	*t*	*β*	*t*	*β*	*t*	*β*	*t*	*β*	*t*	*β*	*t*	*β*	*t*	*β*	*t*	*β*	*t*	*β*	*t*	*β*	*t*
Park land area (m^2^)	−0.00	−0.32	−0.00	−0.09	−0.00	−0.37	0.00	0.10	0.00	0.28	−0.00	−0.05	−0.00	−0.34	−0.00	−0.25	−0.00	−0.30	0.00	0.46	0.00	0.96	−0.00	0.00
Number of parks	0.05	0.06	0.65	1.11	−0.58	−0.44	0.41	0.96	0.55	1.91	0.17	0.25	0.46	1.84	**0.35**	2.00 *	0.52	1.32	0.43	1.80	0.26	1.56	0.53	1.41
Street-network distance to nearest park ^2^ (m)	−0.02	−1.70	−0.02	−1.54	−0.03	−1.35																		
Number of playgrounds	1.09	1.56	0.69	1.41	1.43	1.31	**1.11**	2.30 *	**0.90**	2.69 **	1.16	1.52	**0.78**	2.35 *	**0.52**	2.24 *	0.94	1.81	**0.92**	2.52 *	**0.58**	2.28 *	1.13	1.96
Street-network distance to nearest playground ^2^ (m)	−0.04	−1.81	−0.03	−1.95	−0.04	−1.37																		

Note. Significant results are bolded. Analyses adjusted for gender, age, socio-economic status (highest parental education), GIS-defined walkability (high/low) and clustering of participants within residential neighborhoods (administrative units). ^1^ Out-of-school physical activity. ^2^ Not dependent on buffer size. * *p* ≤ 0.05. ** *p* ≤ 0.01.

**Table 4:** Mixed-model regression results for the relationship between the availability and proximity to parks and playgrounds in the participants’ neighborhood and total physical activity (TPA).

	250 m Street Buffer						500 m Street Buffer						1000 m Street Buffer						1250 m Street Buffer					
	TPA (Per Day)		TPA ^1^ (Weekday)		TPA (Weekend Day)		TPA (Per Day)		TPA ^1^ (Weekday)		TPA (Weekend Day)		TPA (Per Day)		TPA ^1^ (Weekday)		TPA (Weekend Day)		TPA (Per Day)		TPA ^1^ (Weekday)		TPA (Weekend Day)	
	*β*	*t*	*β*	*t*	*β*	*t*	*β*	*t*	*β*	*t*	*β*	*t*	*β*	*t*	*β*	*t*	*β*	*t*	*β*	*t*	*β*	*t*	*β*	*t*
Park land area (m^2^)	−0.00	−0.18	−0.00	−0.81	0.00	0.11	0.00	0.48	−0.00	−0.07	0.00	0.50	−0.00	−0.81	−0.00	−1.21	−0.00	−0.45	0.00	0.56	0.00	1.18	0.00	0.01
Number of parks	0.76	0.33	2.56	1.57	−1.95	−0.52	1.25	1.07	**2.06**	2.58 *	−0.11	−0.06	1.23	1.81	**1.22**	2.52 *	0.89	0.75	1.12	1.76	0.68	1.46	1.40	1.33
Street-network distance to nearest park ^2^ (m)	−0.03	−0.75	−0.05	−1.84	−0.01	−0.11																		
Number of playgrounds	1.48	0.67	**3.00**	2.24 *	−0.72	−0.19	2.33	1.87	**3.08**	3.37 ***	−0.03	−0.01	**2.66**	3.22 *	**2.09**	3.38 ***	2.77	1.78	**2.74**	2.99 **	**2.11**	3.02 **	3.00	1.70
Street-network distance to nearest playground ^2^ (m)	−0.03	−0.49	**−0.08**	−2.14 *	0.02	0.19																		

Note. Significant results are bolded. Analyses adjusted for gender, age, socio-economic status (highest parental education), GIS-defined walkability (high/low) and clustering of participants within residential neighborhoods (administrative units). ^1^ Out-of-school physical activity. ^2^ Not dependent on buffer size. * *p* ≤ 0.05. ** *p* ≤ 0.01. *** *p* ≤ 0.001.

**Table 5:** Mixed-model regression results for the relationship between the availability and proximity to parks and playgrounds in the participants’ neighborhood and active commuting to/from school (ACS) and BMI percentile.

	250 m Street Buffer				500 m Street Buffer				1000 m Street Buffer				1250 m Street Buffer			
	ACS ^1^		BMI Percentile		ACS ^1^		BMI Percentile		ACS ^1^		BMI Percentile		ACS ^1^	BMI Percentile		
	*β*	*t*	*β*	*t*	*β*	*t*	*β*	*t*	*β*	*t*	*β*	*t*	*β*	*t*	*β*	*t*
Park land area (m^2^)	−0.00	−0.22	**−0.00**	−2.51 *	−0.00	−0.71	**−0.00**	−2.24 *	0.00	0.16	−0.00	−0.81	0.00	0.11	0.00	0.51
Number of parks	−0.06	−0.61	−1.38	−1.16	0.02	0.46	0.01	0.02	0.02	0.56	−0.11	−0.27	0.01	0.35	−0.03	−0.08
Street-network distance to nearest park ^2^ (m)	0.00	−0.45	0.00	0.04												
Number of playgrounds	**0.27**	2.85 **	−0.64	−0.58	**0.29**	3.89 ***	0.16	0.20	0.00	0.03	0.43	0.82	−0.01	−0.17	0.35	0.60
Street-network distance to nearest playground ^2^ (m)	0.00	−0.82	0.01	0.16												

Note. Significant results are bolded. Analyses adjusted for gender, age, socio-economic status (highest parental education), GIS-defined walkability (high/low) and clustering of participants within residential neighborhoods (administrative units). ^1^ Analyses was also adjusted for distance to school from participants’ residence. ^2^ Not dependent on buffer size. * *p* ≤ 0.05. ** *p* ≤ 0.01. *** *p* ≤ 0.001.

**Table 6:** Differences in outcomes between participants with high (Q1) and low values (Q4) for significant park and playground attributes.

	Buffer Size	Outcome	Differences in Outcomes between Highest (Q1) and Lowest (Q4) Values of Park and Playground Attributes		
			Q1 vs. Q4 Mean (SD)	Difference between Q1 and Q4	*p*-Value ^2^
Park land area (m^2^)	250 m	BMI percentile	53.23 (28.58) vs. 62.42 (28.01)	−9.19	0.271
		% Overweight/obesity	15.8 vs. 26.9	−11.1%	0.375 ^3^
	500 m	BMI percentile	60.66 (25.60) vs. 65.01 (32.05)	−4.35	0.512
		% Overweight/obesity	20.0 vs. 42.9	−22.9%	0.116 ^3^
Number of parks	500 m	TPA weekday ^1^	165.04 (50.25) vs. 142.83 (28.39)	22.21 min/day	0.119
	1000 m	MVPA weekday ^1^	30.00 (11.61) vs. 29.12 (13.29)	0.88 min/day	0.754
		TPA weekday ^1^	156.55 (49.99) vs. 142.96 (26.20)	13.59 min/day	0.521
Number of playgrounds	250 m	TPA weekday ^1^	162.84 (35.57) vs. 145.25 (30.82)	17.59 min/day	0.054
		ACS	**10.00** (**0.00**) **vs. 6.59** (**4.37**)	**3.41 trips/week**	**0.001**
	500 m	MVPA day	45.21 (13.13) vs. 40.03 (14.64)	5.18 min/day	0.272
		MVPA weekday ^1^	32.07 (13.27) vs. 27.39 (8.19)	4.68 min/day	0.504
		TPA weekday ^1^	169.89 (57.89) vs. 145.26 (28.15)	24.63 min/day	0.420
		ACS	**10.00** (**0.00**) **vs. 4.91** (**4.53**)	**5.09 trips/week**	**0.005**
	1000 m	MVPA day	50.99 (11.44) vs. 41.02 (23.00)	9.97 min/day	0.058
		MVPA weekday ^1^	32.69 (10.52) vs. 28.77 (13.90)	3.92 min/day	0.310
		TPA day	261.29 (45.55) vs. 227.02 (56.86)	34.27 min/day	0.100
		TPA weekday ^1^	157.18 (34.99) vs. 140.46 (24.77)	16.72 min/day	0.161
	1250 m	MVPA day	**44.38** (**13.54**) **vs. 37.25** (**21.55**)	**7.13 min/day**	**0.042**
		MVPA weekday ^1^	30.00 (9.54) vs. 28.19 (14.26)	1.81 min/day	0.411
		TPA day	**259.81** (**42.78**) **vs. 224.26** (**54.67**)	**35.55 min/day**	**0.028**
		TPA weekday ^1^	**164.59** (**41.96**) **vs. 142.28** (**29.99**)	**22.31 min/day**	**0.047**
Street-network distance to nearest playground (m)	-	TPA weekday ^1^	**151.65** (**45.64**) **vs. 165.74** (**28.32**)	**−14.09 min/day**	**0.032**

Note. Significant results are bolded. TPA: total physical activity. MVPA: moderate to vigorous physical activity. ACS: active commuting to/from school. ^1^ Out-of-school physical activity. ^2^ *p*-values from the Mann–Whitney U test. ^3^ *p*-values from the chi-square test.

## Data Availability

The data are not publicly available due to privacy issues.
